# Solidarity and the ethics of exposing others to risk in medical research

**DOI:** 10.1111/bioe.13049

**Published:** 2022-05-16

**Authors:** Paweł Łuków

**Affiliations:** ^1^ Faculty of Philosophy University of Warsaw Warszawa Poland

**Keywords:** exposure to risk, human experimentation, medical knowledge, medical research, solidarity

## Abstract

The ethical justifiability of the invitation of others to participate in research and their deliberate exposure to risks of harm is not a common topic in bioethics. If, however, some offers ought not to be made and the corresponding actions ought not to be facilitated, invitations to, and the conduct of, a medical study involving humans needs justification. This paper addresses this issue by linking the search for medical knowledge with solidarity. The argument begins with the observation that scientific research is aimed at general knowledge, which is a necessary condition of the social value of research. The applicability of this knowledge to many makes it potentially a public good; that is, a good that is available freely to all. For knowledge to be a public good, a social decision to make it freely available to all needs to be made. It is proposed that this decision be grounded in society's, and so in both researchers' and potential research participants’, commitment to solidarity and its obligations of provision, sharing, support, and loyalty. These obligations imply, among other things, an imperfect obligation to participate in research and the corresponding entitlement of the investigators to invite others to participate in research, and so to expose them to its risks during implementation. This entitlement is exercised in an environment shaped by the standards and protections of research ethics and the relevant institutional arrangements.

When invented, new diagnostic and treatment methods make headlines. But headlines that report serious adverse events or the death of a medical research participant seem to attract much more attention. This attention is often accompanied by suspicions of misconduct, which may suggest that its likelihood is considered to be higher in medical research than in other areas. This unease about medical research involving humans is not limited to the media. Evidence of it can also be found in some regulatory documents, including those of the highest rank, when they refer to medical or scientific research in the context of the prohibition of torture or of cruel, inhuman or degrading treatment or punishment. For example, Article 7 of the International Covenant on Civil and Political Rights states that “no one shall be subjected without his free consent to medical or scientific experimentation.” The Constitution of Poland stipulates in article 39 that “No one shall be subjected to scientific experimentation, including medical experimentation, without his voluntary consent.” This concern is not fully accounted for by the memory of the atrocities of World War II (although this did play a key role in the drafting of the Covenant^1^) or by mistrust of “big pharma.”

The reason for concern may lie in the very idea of experimentation on humans involving risk of harm to its participants, as is the case in a significant proportion of medical research. In every experiment involving risk of harm to its participants, the researcher invites potential participants to accept that risk, and, when they decide to participate, deliberately exposes them to it by implementing the study. Thus, what may cause concern about medical experimentation involving risk to its participants is the intuitive recognition that invitations of competent (i.e., capable to give informed consent) others to take risk, and their deliberate exposure to it, require ethical justification. Perhaps it is this lack of a convincing and socially acceptable justification of such invitations and exposures that explains the uneasiness about research involving humans, despite the enthusiasm for medical innovation, which is clearly explained by the need to protect human life and restore health.

Such a justification is not easy. It cannot rest on narrowly consequentialist thinking that invokes the worthy goal of obtaining knowledge. First, the ethics of research involving humans is based on the rejection of such a narrowly consequentialist perspective, as evidenced by the various ethical standards and protections of research participants, such as, among others, the requirements of minimization of risk, or approval of the research proposal by a research ethics committee.^2^ Second, the knowledge gained in research or the benefits from the intervention being investigated cannot justify the deliberate exposure of competent others to risks, because the very need to conduct research shows that at the time of invitation to participate neither that knowledge nor those benefits are known to be available in the study or as its result. Third, the knowledge obtained in a medical study frequently does not benefit those who are exposed to its risks. Encouraging competent others to participate in undertakings such as medical research, which expose them to risk of harm, needs a stronger justification than expected beneficial results.

Nor is a sufficient justification to be found in informed consent alone (volenti non fit iniuria), where participation in medical research is seen as a matter of free exercise of individual rights by a competent person. On this view, a potential participant accepts risks of harm and, aside from the problem of therapeutic misconception, expects benefits (for themselves or for others), while the investigator provides the participant with adequate information on possible benefits and risks related to the study and, if appropriate, makes the potential benefits available to them. However, it is clear that informed consent can justify the conduct of a study involving a human subject, but not the making of the offer to participate in it. It is based on the assumption that the offer is beyond reproach. And this is exactly what needs to be established.

Common moral awareness suggests that certain *offers ought not to be made* and that involvement of competent individuals in respective activities *ought not to be deliberately facilitated*. Examples of such offers or facilitations are numerous and their gravity varies. One class comprises “indecent proposals.” Theft, prostitution, and betrayal are intuitive examples of actions which we ought not to encourage others to undertake, or assist them in their performance, because they are seen as wrong. Another class of such acts are “excessive expectations” to engage in acts that, although not criticizable and often admirable, ought not to be suggested to others or, more disputably, facilitated. Supererogatory acts belong to this class. Encouragement or invitation to engage in acts of heroism or self‐sacrifice can be perceived as unjustified because they are excessive or a form of undue pressure, even if they are expected to produce significant benefits or goods and the agent is competent and aware of the risks of involvement. A third class of acts that can be seen as those that ought not to be encouraged or facilitated are the objects of “offers you can't refuse,” whatever the motives of the person who makes the offer. These actions can involve deliberate victimization in exploitative practices, or the involvement of persons who are in despair or deprived of alternatives to activities that they would otherwise avoid or not even consider.

These examples are not intended to suggest a kind‐identity of invitation to, or facilitation of, acts of these classes with the invitation of competent others to participate in, and implementation of, medical research. Although perhaps not as precisely as one might wish, the examples show simply that some offers, ethically speaking, must not be made even if they are accompanied by certain protections. Assurances that a solicited theft is of small scale, that an act of sacrifice by the would‐be hero will be appreciated ex post, or that a loss of self‐respect will be temporary, do not justify the making of such offers. Similarly, if invitations of competent others to participate in medical research, involving intentional exposure to risks of harm by participation, are not to be deemed indecent proposals, excessive expectations, or offers that cannot be refused, deliberate exposure of others to risks of harm in medical research needs convincing justification.

It will be argued below that such justification can be provided by a shared commitment to solidarity on the part of both investigators and potential and actual participants. A shared commitment to solidarity allows some of us to invite competent others to participate in undertakings in which they will be deliberately exposed to risks of harm in order for the benefits that result from such exposure to be shared potentially with all. The promotion of a commitment to solidarity may also help reduce the uneasiness caused by human experimentation.

The argument that follows will start with the observation that scientific research is aimed at knowledge, which is a necessary (although not the sole) condition of the social value of research. The applicability of this knowledge to many makes it potentially a public good; that is, a good that is available freely to all. Next, it will be argued that the perception of knowledge as a public good can be founded on the commitment of society, including both investigators and potential research participants, to solidarity. A sketch of solidarity as an aspect of the organization of society will identify the key obligations of solidarity. On this ground, an imperfect obligation of a society's members to participate in research will be identified, together with the corresponding entitlement of the investigators to invite competent others to participate in research, and so expose them to its risks.

## 1 SOCIAL VALUE OF RESEARCH AND KNOWLEDGE

The requirement of the social value of research has been present throughout the modern era, in one form or another, in the standards of research ethics involving humans. The Nuremberg Code requires that “The experiment should be such as to yield fruitful results for the good of society.”^3^ Similar provisions are included in various international and national guidelines on the ethics of research involving humans. The Declaration of Helsinki stipulates that “Medical research involving human subjects may only be conducted if the importance of the objective outweighs the risks and burdens to the research subjects.”^4^ The Council for International Organizations of Medical Sciences (CIOMS) Commentary on Guideline 1 states that “In order to be ethically permissible, health‐related research with humans, including research with samples of human tissue or data, must have social value.”^5^ The U.K.'s *Governance arrangements for research ethics committees: 2020 edition* requires that the exposure of research participants to the risks, burdens and intrusions of research must be “justified by the expected benefits for the participants or for science and society.”^6^ There is also consensus among scholars that the social value of research involving humans is a precondition of its ethical acceptability.^7^

Precise conceptualization of the social value of a study is difficult, in particular in relation to its scientific validity.^8^ The concept of social value is variously used and plays multiple roles in the ethics of medical research involving humans.^9^ However, being one of the central ethics requirements related to research involving humans, it is also clearly linked to the risks of harms to, and to the expected benefits for, the study's participants. Two things deserve special attention in this context. First, however harms and benefits are understood, the central contrast, at least at the linguistic level, is that between harms and benefits to *individuals* and the value to *society*. It is individuals who are exposed to the risks associated with their participation in a study and who may benefit from being included. The social value of the study is typically seen as accruing, collectively, to society, in the form of anticipated benefits stemming from the knowledge gained in medical research or of the value of knowledge.^10^ Unless one ascribes real existence to society as distinct from the individuals of whom it is composed, the benefits or value to society cannot be seen in abstraction from those individuals, who can obtain benefits. Thus, any view of the social value of research must include the value or benefits that can be expected from a particular study by the members of society who do not participate in that study.

Secondly, the link between potential harms and benefits for participants and the potential value or benefits for society is knowledge that applies not only to the participants but also to other members of society—all of them or a subpopulation. Such knowledge can potentially contribute to explanations of social, psychological, or health‐related phenomena. In this sense, research may generate knowledge valued for its own sake. It can also be valued instrumentally, as a potential basis for the development of technologies that can alleviate problems affecting members of larger groups of individuals or the whole society, and so it may generate tangible benefits for many or perhaps all. By contrast, a study that produces knowledge pertaining only to one person cannot be regarded as being of value or benefit to society.

Thus, study participants are the necessary means to knowledge.^11^ The route from sample data to knowledge, namely the process of generalization, can take various forms and depends on the methodology and design of the research. With regard to empirical investigations, as in the life sciences, the route to generalization from sample data to a general claim involves inductive inference, specifically statistical inference. Thus, the standard of the social value of medical research requires that if this route is reliable, a medical study involving humans can be ethically acceptable, and so the exposure of participants to risks of harm can be justified by the study's potential to arrive at, or significantly contribute to, general claims.

On its own, the knowledge or data on an individual study participant does not have the potential of generalizability. What turns data obtained in an investigation into knowledge is the study design, the combination of the data obtained from many participants, and the researchers' work. Data from an individual can become knowledge within the collaborative and structured effort of participants and researchers. In this way, knowledge, which is applicable to many (the whole population or a subsection of it), emerges from structured social collaboration. A study's potential to produce knowledge that is valuable (for its own sake or for the benefits that can derive from its application) to society, and that is a necessary constituent of the social value of that study, is thus a result of a structured collaboration of participants and researchers.

The generation of knowledge that is applicable to many, which is the goal of an investigation, can be justified epistemically by reference to the values of truth, reliability, the progress of science, and so forth. However plausible such justifications are, these epistemic values do not provide a sufficient ethical justification for the intentional exposure by the investigators of competent individuals to risks of harm by participation in medical research. First, the standard of the primacy of the human being subordinates these epistemic values, and the pursuit of knowledge, to other values. It prohibits the exposure of participants to excessive risks of harm and mandates balancing such risks against expected benefits of research. The pursuit of knowledge is not the supreme or the only foundation of the ethics of medical research involving humans. Respect for autonomy, which is closely related to the primacy of the human being and which can be seen as one of the main moral limits on the pursuit of knowledge, is not a sufficient justification for exposing competent others to risks of harm from research either. If it were, the various requirements of informed consent—such as provision of knowledge, absence of undue influence, and, in particular, the ethical oversight of research—would not belong to the core of the ethics of medical research involving humans.

To identify the value that justifies the intentional exposure of research participants by researchers to the risks of harms, the two considerations mentioned above need to be related to the generalizability of the data obtained, which makes the knowledge resulting from them a public good. A public good, as it is standardly understood in economics, is a good that, once produced, is freely available to all and does not diminish with use.^12^ Unless measures intended to diminish access to it are undertaken, general knowledge can become, and be perceived as, a good for every member of society. Perceived as a public good, knowledge and, to a lesser extent, the results of the technologies, remedies and so forth developed on its basis, can be shared with, and become beneficial to, those who did not or do not contribute to its production. In this respect, knowledge is analogous to the things that are characterized in international law as “the common heritage of humankind” and which are not to be used exclusively for the benefit of some but are to be preserved for posterity.

This view of knowledge as a socially shared good is clearly idealistic. In the real world, the growing economic potential and value of knowledge, large private investments in medical research and its commodification,^13^ corporate secrecy, patenting and copyright protections all challenge the perception of knowledge as a public good.^14^ However, the number and extent of the phenomena that encourage scepticism about knowledge as a public good is rather recent,^15^ and the entities responsible for limiting access to knowledge for financial reasons alone are often criticized. Thus, the idea of knowledge as a public good is in principle plausible. In the present context, it can be conceived of as an ideal type that is to structure thinking about the intentional exposure of competent others to the risks of participation in research. It can guide conceptualization of the social value of research and justification of the intentional exposure of participants to risks.

Goods can be public in virtue of their nature (e.g., the atmospheric air, which is available to everyone) or through intentional arrangements (such as subsidies for public transport, which benefit both city inhabitants and visitors, who do not pay local taxes). Thus, a decision can be made whether a particular good is public or not. As restrictions imposed on access to knowledge show, scientific knowledge can, if appropriate means for its storage and transmission are available, be a public good, depending on the decisions of individuals or society as a whole (e.g., in the form of regulation). It can be a public good if we decide so. Because such decisions need to be based on values, for knowledge obtained as a result of experimentation to be a public good (in a society or globally), an appropriate, socially recognized value needs to be identified. This value could be seen as providing investigators with justification for their invitation of competent persons to participate in medical research and for exposing them to the risks of harm by participation.

It will be proposed that the value in question is solidarity, which relies on sharing goods and services among members of a group independently of their individual contributions to their production but with recognition of those who made these contributions.

## 2 PRACTICES AND OBLIGATIONS OF SOLIDARITY

The concept of solidarity seems to elude attempts at definition. Its history, while long, is marked by gaps and changes in the meaning of the term. Sometimes it is understood as a descriptive concept and at other times as normative.^16^ Scholarly treatments of solidarity, whether descriptive or normative, frequently do not define the concept, and the treatments that do are not mutually equivalent, sometimes being contradictory.^17^ Solidarity has been understood as, among other things, a recognition of common interests, a cause or goal,^18^ a mechanism of social cohesion grounded on the division of labor,^19^ a sense of belonging to a group or culture,^20^ shared social ideals,^21^ a shared form of collective life,^22^ and empathy.^23^

A comprehensive discussion of the idea and value of solidarity lies beyond the scope of this paper. The account of solidarity to be presented in this section is a normative proposal, intended to explain what it means to see the knowledge obtained in research involving humans as a public good. In effect, solidarity will be seen as the ground of justification of the deliberate exposure by investigators of competent persons to risks of participation in research.

Solidarity, it will be proposed, is a normative concept that underlies various attitudes and practices relating to the exposure to risks and burdens for the benefit of unspecified others who are partners in collaborative undertakings. The central idea of this normative concept is the recognition of the need for transfer of benefits, which have been obtained at a cost (including risks and burdens) to their provider without demanding compensation from a potential or actual beneficiary within a collaborative endeavor. Solidarity construed in this way is a moral value that gives direction to individual decisions and actions, and structures social practices and institutional arrangements, rather than  a merely moral‐political obligation^24^ or a sense of feeling with others.^25^

Solidarity, as it is to be understood here, needs to be distinguished from altruism. It does not demand selfless sacrifice or devotion to the interests of others. Rather, it links self‐interest with the interests of unspecified others within a collaborative undertaking, which presupposes recognition of vulnerability, dependence, and the mutuality of collaborators. Such a collaboration is not necessarily demanded by justice. Arguably, arrangements based on solidarity complement those of justice, and may compensate for the deficiencies of individualism and the market. Thus, it is linked to the idea of membership in a group of vulnerable, dependent collaborators.

Construed as a moral value, solidarity grounds obligations that can bind individuals involved in the activities and institutions that deliver goods or services. Such obligations of solidarity include: the obligation of *provision* of, or contribution to the procurement of, a good or service that is to be made available to all; the obligation to *share* the good according to a standard that does not require maximization of compensation for the costs or burdens related to the production of this good; the obligation of *support* to provide for those in need of the good or service despite an absence of reasons to expect reciprocation; and the obligation of *loyalty* to sustain the cooperative relationship between providers and recipients of the good or service, even if more attractive alternative options to allocate the good or service are available.^26^

These obligations can bind on the interpersonal and agent level or on the system level.^27^ When considered as moral obligations of individuals, they are—to use the traditional term—imperfect obligations,^28^ in that it is up to the agent, who endorses the value of solidarity, to decide when to discharge them and who in particular will be the beneficiary of their actions or undertakings, that is, with regard to who in particular the four obligations of solidarity will be discharged. On the system level, when incorporated in regulations, these obligations specify, more narrowly, the classes of individuals required to perform them and the classes of those to whom these obligations are owed. In this way, on the system or regulatory and institutional level, these obligations cease to be imperfect and do not leave equally large room for the choice of their beneficiaries, as it is the case with moral imperfect obligations of individuals.

Thus, as a feature of collaborative undertakings (such as public healthcare or the pursuit of knowledge), solidarity will be understood as a desirable state of social arrangements in which certain services or goods are seen as public goods that are provided by some who undertake risks or bear burdens for the benefit of unspecified others, and in which these goods, or the results of their use, are made available to those others without demanding compensation from them.^29^

## 3 FOR THE SAKE OF SOLIDARITY: EXPOSING COMPETENT OTHERS TO RISKS

Solidarity, understood as explained above, encourages the perception of knowledge that is obtained in research as a public good. It requires that the knowledge, which could be used to the exclusive advantage of those who produce it, be used for the benefit of those who have not participated in its generation or cannot be said to have a proprietary claim to it or to the potential technologies developed on its basis. In consequence, the recognition of the value of solidarity in relation to the production of medical knowledge generates the four obligations of solidarity: to provide and share knowledge, or the results of its application, with others when they need it and in response to that need.

Potential participants who endorse solidarity will recognize the obligation to contribute to the provision of the public good of medical knowledge. In consequence, they will be open to requests to participate. Since response to a request of that kind is conditional on the willingness of the person approached to contribute to the production of the public good on a given occasion, the obligation to participate in a study, namely the obligation to collaborate in the enterprise of the generation and sharing of knowledge, or in the results of its application, with other members of the society who need it, can only be imperfect. Participation in research can be requested but it must not be demanded. It is up to the person in question to decide how they will respond to a request of that kind; that is, whether they will contribute to the development of knowledge on a given occasion. In parallel, reciprocation can be expected and asked from others, but it cannot be justifiably demanded from particular individuals who benefited from that knowledge or its application.

Recent large‐scale coronavirus vaccine field trials provide an apt illustration of the role of solidarity understood as relying on an imperfect obligation to participate in research. These placebo‐controlled trials involved thousands of individuals who volunteered to participate in research of immense potential social value. They could not be guaranteed to receive a candidate vaccine, and it was unknown whether the vaccine would give them protection against the virus. Assuming that they were properly informed about the risks and potential benefits of participation, that they were not unusually risk‐seeking, and did not suffer excessively from therapeutic misconception, their decision to participate in the trials cannot be explained solely by the pursuit or expectation of benefits to themselves. Although such a thesis would require empirical confirmation, one can hypothesize that by exposing themselves to the virus without sufficient assurance of protection against infection, the volunteers decided to discharge their imperfect obligations of solidarity to contribute to the provision of a public good, share this good with others who are in need of support, and thereby sustain a cooperative relationship.

This context of imperfect obligations of solidarity does not necessarily call into question payments to research participants as recruitment incentives. If the obligation to participate were construed as a perfect obligation, such payments would not be justified. By contrast, the imperfect character of the obligations of solidarity can leave room for incentives to enrolment, if they do not (and are not intended to) compensate for the burdens of participation, and so do not undermine the ethical ground of solidarity.

To further elucidate the consequences of the perception of medical research involving humans as contextualized by a social commitment to solidarity, one can ask what happens if solidarity is removed from the picture and how far it can be removed. Without the normative context of solidarity construed as relying on imperfect obligation, the knowledge obtained in medical research will tend to be seen, similarly to the knowledge produced in industrial science,^30^ as a private good, which can be claimed only by those who have contributed to its production and which can be delivered for a fee to those who have not produced it. Although participation in research can be ethically justified in such a framework on the condition of remuneration to participants or their sharing in the benefits from the knowledge obtained, the invitation to participate and the deliberate exposure of others to the risks of research would still need a justification, which is not available in a solidarity‐free context. The required justification, as it is proposed here, is furnished by the normative context of solidarity.

It can be objected that the view of solidarity as involving the four imperfect obligations of provision, sharing, support and loyalty is vulnerable to the free‐rider problem.^31^ If no one in particular must discharge the obligations of solidarity on a particular occasion, it is quite likely that no one will. For solidarity to be not only a cherished value but also a social reality, in which the free‐rider problem is to be overcome or at least sufficiently attenuated, a network of practices and institutions of solidarity is required. Among others, there needs to be an environment of fair distribution of benefits and burdens of social interaction, which rely on attitudes, motives, and actions that are sufficiently pervasive and effective to ground mutual trust.^32^ Such a complex of attitudes, motives, and actions, which can be collectively called the environment of mutual concern, can bring about a sense of belonging with others. This belonging will be accompanied by empathy and recognition of others as collaborators or co‐members in one society. In order for them to be motivated to act or take risks for the benefit of others who are in need, society members need to appreciate their own and others' vulnerability and dependence. To respond to vulnerability and dependence, members of society will need to be committed to mutuality and reciprocation, which in turn requires a minimal level of reliability and loyalty.

It might be thought that this approach to solidarity presupposes strong communal bonds (among fellow citizens, group members, social class members, humanity as a whole, etc.), which require deep commitment to shared values or goals, as well as attachment to identifiable others who share this commitment.^33^ Clearly, if solidarity were to be based on a rich comprehensive moral view, it could afford justification of the intentional exposure of others to risks of participation in medical research only among those who share that particular view. In effect, the knowledge produced in medical research could not be viewed by them as a public good.

This is a serious concern. Contemporary societies are diverse. Their members endorse divergent comprehensive views; citizens of liberal societies value individuality and pursue different life goals; the economies of such societies rely on markets driven predominantly by self‐interest. Even deeper differences divide states and regions. A rich comprehensive doctrine, which included commitment to shared political principles and values,^34^ would not provide universally compelling reasons to justify the intentional exposure of others to risks of participation in medical research. Not all societies share sufficiently congruous commitments, and those that do, do not seem to be united by one political vision. If the concept of solidarity is to be useful in the context of the pursuit of knowledge—the good produced in medical research involving humans and that is to be seen as the common heritage of humanity—it must not require a rich comprehensive view of human life and the goals of society.

In order to respond to such worries and provide a plausible justification for the intentional exposure of others to risks of participation in research, the account of solidarity offered above needs to be supplemented with answers to two key questions. First, it has to be identified what in modern diverse societies, or globally, can take the place of the foundation of solidarity and suffice as justification of the intentional exposure of others to risks of participation in medical research. Secondly, it has to be elucidated how the environment of mutual concern, in which medical research involving humans is seen as a practice of the generation and distribution of a public good, can be brought about in such diverse societies, or globally.

The solidarity that is to provide the required justification for the intentional exposure of others to risks of participation in medical research intended to provide universally applicable knowledge must rely on the appreciation of goods or goals that are recognized and valued for their own sakes or instrumentally by practically all human beings. These goods or goals need to be perceived as the focus of the collaborative efforts that make up medical research.

In the context of medical research involving human participants, the obvious candidates for the (practically) universally valued goods or goals are human health and life, for the sake of which, in the face of illness or premature death, medical knowledge is pursued. This is not to say that the improvement or restoration of health and the prevention of premature death must be the only goals that unite potential and actual researchers, research participants, or beneficiaries of medical research. Nor is this to say that these goods are the only goods pursued in medical research. Being universally recognized as goods, health and life are to be seen as grounds of the solidarity which justifies the collaborative pursuit of knowledge concerning human health and disease. Members of various groups can be committed to other values. However, health and life must be recognized and endorsed by all human beings, at least to the extent to which health and life are preconditions of all other human pursuits. In consequence, the universal commitment to the values of human health and life in the face of such adversities as illness or premature death can inspire a sense of “being in this together,” and so can motivate the pursuit of knowledge in medical research involving humans. The quest for medical knowledge needs collaborative undertakings and arrangements that can encourage attitudes, motives, and actions that together make up the environment of mutual concern.

Such undertakings and arrangements are not regulated by a strong obligation to sacrifice for the benefit of others, nor by relations based exclusively on supply and demand. They are structured by a commitment to collaboration in an undertaking that is a systematic response to the recognition of human vulnerability, dependence, and mutuality in the context of the pursuit of the goals of health and life. Medical research is to be seen as an element of a collaborative undertaking, which relies on the obligations of provision and sharing of a good (i.e., knowledge), which is appreciated by (practically) all as instrumentally required for the sake of human health and life, and support of, and loyalty to, all others, who also recognize and appreciate this good at least as an instrumental good. In consequence, participation in such an endeavor obligates its collaborators to contribute to the pursuit of knowledge in order to share it with others without demanding compensation. Participation in this endeavor requires that, in mutual recognition of human vulnerability and dependence, its results be perceived as a public good, which ought to be provided to those who need it, rather than only to those who deserve or can afford it. The invitation of others to participate in research is an encouragement to the performance of the imperfect obligations of human solidarity.

## 4 CONCLUSION

The purpose of this paper was not to suggest that solidarity is to be seen as the backbone of all or most collaborative social practices. Its focus was on the kind of medical research whose goal is the production of knowledge that is applicable and (potentially) beneficial to all. Although it is likely that solidarity can ground all collaborative endeavors in which public goods are sought, and in many other social undertakings, no attempt has been made to show if and which other areas of social life can be conceptualized in this way.

The argument offered above justifies investigators' exposure of competent participants in medical research to risks of harm by linking the quest for medical knowledge with solidarity. It sees medical research as part of a larger enterprise of the pursuit of the universally recognized goods of human life and health in the face of premature death and illness. Solidarity encourages the perception of knowledge, which is sought in such research, as a public good; that is, as a good to be shared with those who did not contribute to its production and without demanding a fee from them. Solidarity also requires that society members recognize the imperfect obligation to contribute to the provision of this public good. It is in response to this obligation that researchers can be entitled to invite others to contribute by participating in research, avoiding in this way indecent proposals, excessive expectations, or offers that cannot be refused. This entitlement is exercised in the environment of the various standards and protections of research ethics and the corresponding institutional arrangements. These standards, protections, and arrangements do not justify the exposure of competent others to the risks of harm from participation. They guide the process of invitation to participate and the conduct of research. Rooted in solidarity, they contribute to the environment of mutual concern, in which the social perception of medical research involving humans can be in line with the enthusiasm for medical innovation.

## CONFLICT OF INTEREST

The author declares no conflict of interest.

## Footnotes

1 Nowak, M. (2005). *U.N. Covenant on civil and political rights: CCPR commentary* (2nd rev. ed., pp. 188‐192). N.P. Engel.

2 This, for some, makes research ethics deserve the label of paternalism; for example, Miller, F. G., & Wertheimer, A. (2007). Facing up to paternalism in research ethics. *Hastings Center Report*, *37*(3), 24–34. https://doi.org/10.1353/hcr.2007.0044; Jansen, L. A., & Wall, S. (2009). Paternalism and fairness in clinical research. *Bioethics*, *23*(3), 172–182. https://doi.org/10.1111/j.1467-8519.2008.00651.x

3 The Nuremberg Code. (1949). *Trials of war criminals before the Nuremberg Military Tribunals* (pp. t. II, s. 181–182). U.S. Government Printing Office.

4 World Medical Association (WMA). (2013). *WMA Declaration of Helsinki*—*Ethical principles for medical research involving human subjects*. Fortaleza, Brazil, October 2013.

5 Council for International Organizations of Medical Sciences (CIOMS). (2016). *World Health Organization: International ethical guidelines for health‐related research involving humans.* Geneva 2016.

6 National Health Service (NHS). (2020). *Governance arrangements for research ethics committees: 2020 edition. 1.2.2*. Retrieved from https://www.hra.nhs.uk/media/documents/GAfREC_Final_v2.0_26.03.2020.pdf

7 Casarett, D. J., Karlawish, J. H., & Moreno, J. D. (2002). A taxonomy of value in clinical research. *IRB*, *24*(6), 1–6.; Emanuel, E. J., Wendler, D., & Grady, C. (2000). What makes clinical research ethical? *Journal of the American Medical Aassociation*, *283*(20), 2701–2711. https://doi.org/10.1001/jama.283.20.2701; Freedman, B. (1987). Scientific value and validity as ethical requirements for research: A proposed explication. *IRB: Ethics & Human Research*, *9*(6), 7–10.

8 Emanuel, E. J., Wendler, D. D., & Grady, C. C. (2008). Ethical framework for biomedical research. In E. J. Emanuel, C. C. Grady, R. A. Crouch, R. K. Lie, F. G. Miller & D. D. Wendler (Eds.), *The Oxford textbook of clinical research ethics* (pp. 123–135). Oxford University Press.

9 Habets, M. G., van Delden, J. J., & Bredenoord, A. L. (2014). The social value of clinical research. *BMC Medical Ethics*, *15*, 66. https://doi.org/10.1186/1472-6939-15-66; Ganguli‐Mitra, A., Dove, E. S., Laurie, G. T., & Taylor‐Alexander, S. (2017). Reconfiguring social value in health research through the lens of liminality. *Bioethics*, *31*(2), 87–96. https://doi.org/10.1111/bioe.12324

10 Habets et al., op. cit. note 9.

11 Emanuel et al., op. cit. note 8, p. 125.

12 Head, J. G. (1972). Public goods and public policy. In C. K. Rowley (Ed.), *Readings in industrial economics. Vol. 2: Private enterprise and state intervention* (pp. 66–87). Macmillan Education U.K.; Olson, M. (1971). *The logic of collective action: Public goods and the theory of groups*. Harvard University Press; Stiglitz, J. (1999). Knowledge as a global public good. In I. Kaul, I. Grunberg & M. Stern (Eds.), *Global public goods: International cooperation in the 21st century*. Oxford University Press.

13 Radder, H. (2010). *The commodification of academic research: Science and the modern university*. University of Pittsburgh Press.

14 Dalrymple, D. (2003). Scientific knowledge as a global public good: Contributions to innovation and the economy. In J. Esanu & P. Uhlir (Eds.), *The role of scientific and technical data and information in the public domain: Proceedings of a symposium*. National Academies Press; Machlup, F. (1980). *Knowledge: Its creation, distribution and economic significance. Vol. 1, Knowledge and knowledge production*. Princeton University Press.

15 Ziman, J. M. (2000). *Real science: What it is, and what it means*. Cambridge University Press.

16 Bayertz, K. (1999). Four uses of “Solidarity”. In K. Bayertz (Ed.), *Solidarity* (pp. 3–28). Springer Netherlands.

17 For an overview of clarifications of the concepts, see chapter 5 of Zoll, R. (2000). *Was ist Solidarität heute?* (1. Aufl. ed.). Suhrkamp.

18 Engels, F. (1975). On the history of the communist league. In *Karl Marx, Frederick Engels: Collected works* (R. Dixon et.al, Trans.) (Vol. 26, pp. 312–330). Lawrence and Wishart.

19 Durkheim, E. (1984). *The division of labor in society* (W. D. Halls, Trans.). Free Press.

20 Rorty, R. (1989). *Contingency, irony, and solidarity*. Cambridge University Press.

21 Brunkhorst, H. (2005). *Solidarity: From civic friendship to a global legal community* (J. Flynn, Trans.). MIT Press; Kolers, A. (2016). *A moral theory of solidarity* (1st ed.). Oxford University Press.

22 Habermas, J. R. (1991). Gerechtigkeit und Solidarität. In *Erläuterungen zur Diskursethik* (1. Aufl. ed., pp. 49–76). Suhrkamp.

23 Bartky, S. L. (2002). Sympathy and solidarity. In S. L. Bartky (Ed.), “*Sympathy and solidarity” and other essays* (pp. 69–90). Rowman & Littlefield.

24 Kolers, op. cit. note 21.

25 Bartky, op. cit. note 23.

26 Tranow, U. (2012). *Das Konzept der Solidarität: Handlungstheoretische Fundierung eines soziologischen Schlüsselbegriffs* (pp. 63–73). Springer VS.

27 Ibid.

28 Kant, I. (1996 [1785]). Groundwork of the metaphysics of morals. In M. J. Gregor (Ed.), *Practical philosophy*. The Cambridge edition of the works of Immanuel Kant (pp. 37–108). Cambridge University Press; Mill, J. S. (1965). Utilitarianism. In J. B. Schneewind (Ed.), *Mill's ethical writings* (1st ed.). Collier Books; Pufendorf, S. (1994). On the law of naure and of nations in eight books. In M. J. Seidler (Ed.), *The political writings of Samuel Pufendorf.*(Trans. C. L. Carr). Oxford University Press.

29 Tranow, op. cit. note 26.

30 Ziman, op. cit. note 15.

31 Elster, J. (1989). *The cement of society: A study of social order*. Cambridge University Press.

32 Baier, A. (1986). Trust and antitrust. *Ethics*, *96*(2), 231–260; Hollis, M. (1998). *Trust within reason*. Cambridge University Press.

33 Capaldi, N. (1999). What's wrong with solidarity? In K. Bayertz (Ed.), *Solidarity* (pp. 39–56). Springer Netherlands; Engelhardt, H. T., Jr. (1999). Solidarity: Post‐modem perspectives. In K. Bayertz (Ed.), *Solidarity* (pp. 293–308). Springer Netherlands; Rorty, op. cit. note 20.

34 Brunkhorst, op. cit. note 21; Taylor, C. (1989). Cross‐purposes: The liberal‐communitarian debate. In N. Rosenblum (Ed.), *Liberalism and the moral life* (pp. 159–182). Harvard University Press.

